# Effects of Body Weight Strength Training on Cognitive Function and Quality of Life in Healthy Older People: A Systematic Review of Randomized Controlled Trials

**DOI:** 10.3390/life15111698

**Published:** 2025-11-02

**Authors:** Álvaro Levín Catrilao, Bastián Parada-Flores, Pablo Aravena-Sagardia, Edgar Vásquez-Carrasco, Jordan Hernandez-Martinez, Felipe Caamaño-Navarrete, Carlos Arriagada-Hernandez, Cristian Sandoval, Tomás Herrera-Valenzuela, Braulio Henrique Magnani Branco, Pablo Valdés-Badilla

**Affiliations:** 1 Doctoral Program in Physical Activity Sciences, Faculty of Education Sciences, Universidad Católica del Maule, Talca 3460000, Chile; alvaro.levin7@gmail.com (Á.L.C.); paradafloresbastian@gmail.com (B.P.-F.); 2 Escuela de Nutrición y Dietética, Facultad de Salud, Universidad Santo Tomás, Talca 3460000, Chile; 3 Physical Education Career, Faculty of Education, Universidad Autónoma de Chile, Temuco 4780000, Chile; pablo.aravena@uautonoma.cl (P.A.-S.); felipe.caamano@uautonoma.cl (F.C.-N.); carlos.arriagada@uautonoma.cl (C.A.-H.); 4 School of Occupational Therapy, Faculty of Psychology, Universidad de Talca, Talca 3465548, Chile; edgar.vasquez@utalca.cl; 5 Centro de Investigación en Ciencias Cognitivas, Faculty of Psychology, Universidad de Talca, Talca 3465548, Chile; 6 Department of Physical Activity Sciences, Universidad de Los Lagos, Osorno 5290000, Chile; jordan.hernandez@ulagos.cl; 7 Department of Education, Faculty of Humanities, Universidad de La Serena, La Serena 1700000, Chile; 8 Escuela de Tecnología Médica, Facultad de Salud, Universidad Santo Tomás, Los Carreras 753, Osorno 5310431, Chile; cristian.sandoval@ufrontera.cl; 9 Departamento de Medicina Interna, Facultad de Medicina, Universidad de La Frontera, Temuco 4811230, Chile; 10 Department of Physical Activity, Sports and Health Sciences, Faculty of Medical Sciences, Universidad de Santiago de Chile (USACH), Santiago 9170022, Chile; tomas.herrera@usach.cl; 11 Graduate Program in Health Promotion, Cesumar University (UniCesumar), Maringá 87050-900, Brazil; braulio.branco@unicesumar.edu.br; 12 Department of Physical Activity Sciences, Faculty of Education Sciences, Universidad Católica del Maule, Talca 3460000, Chile; 13 Sports Coach Career, Faculty of Life Sciences, Universidad Viña del Mar, Viña del Mar 2520000, Chile

**Keywords:** cognition, mental health, resistance training, exercise, aging

## Abstract

Objective: This systematic review evaluated the effects of body weight strength training (BWST) on cognitive function and health-related quality of life (HRQoL) in apparently healthy older people compared with active/inactive control groups. Methods: A literature search was conducted in six databases: PubMed, Web of Science, Scopus, ScienceDirect, EBSCOhost, and PsycINFO. The PRISMA, TESTEX, RoB 2, and GRADEpro tools were used to assess the methodological quality and certainty of evidence. The protocol was registered in PROSPERO (code: CRD42024623038). Results: Among the 27,241 records, 9 randomized controlled trials involving 682 (85% women) apparently healthy older people were included. Compared with the active/inactive control groups, the BWST resulted in significant improvements in orientation to place, language, visuospatial ability, processing speed, attention, and short-term memory. Within-group improvements were observed in general cognitive function and health-related anxiety scores in both the BWST and active control groups. The calculation and set shifting improved only within the BWST group. Conclusions: The individual results of the RCTs suggest that BWST may have potential effects on cognitive function and HRQoL in apparently healthy older people. Nevertheless, the certainty of evidence was insufficient to support definitive recommendations. Therefore, further high-quality studies are needed to establish solid conclusions.

## 1. Introduction

Cognitive function refers to mental processes that enable humans to perceive, interpret, and interact with their environment, involving multiple cognitive domains, which are susceptible to decline with advancing age [1,2,3], which may lead to changes in motor function and increased fall risk in physically inactive older people [4,5]. In contrast, meeting international physical activity recommendations, which suggest between 150 and 300 min of moderate-intensity physical activity or between 75 and 150 min of vigorous-intensity physical activity per week [6], would lead toward a physically active and healthy lifestyle during aging [7,8]. Significant benefits in cognitive function, mainly in processing speed, memory, non-verbal reasoning, problem solving, orientation, short-term memory, and attention [9,10,11,12], and in core components of executive functions (i.e., working memory, inhibitory control, and cognitive flexibility) are observed in apparently healthy older people [13], which are critical for functional independence in activities of daily living and improved health-related quality of life (HRQoL) [14].

In this context, several physical activity strategies such as cardiorespiratory fitness [13], strength training [9,15], mind–body training [16] and multicomponent training [17] have been shown to be effective non-pharmacological treatment interventions to counteract cognitive decline and improve mental health [11]. Their benefits are largely attributed to the modulation of neurotransmitter activity and the upregulation of neurotrophic factors, which promote synaptic plasticity, neuro-genesis, and cell survival [11]. A meta-analysis conducted by Gallardo-Gómez et al. [15] revealed that low doses of strength training (529 metabolic equivalent task METs-min; 3 sessions of 40 min without considering warm-up and cool-down) per week could significantly improve cognitive function compared with other training methods in apparently healthy older people. Furthermore, minimal equipment strength training, such as that using elastic bands [18,19] or body weight exercises [20], provides adaptable and accessible options, obtaining benefits mainly in muscle strength, which could be an important physical fitness marker in age-related cortical and subcortical neurodegeneration with implications in specific cognitive domains, such as spatial working memory, cognitive flexibility [21], executive function and global cognitive function, in apparently healthy older people [22].

Similarly, loss of muscle strength in the upper and lower body increases the probability of presenting a low HRQoL in apparently healthy older people [23]. This multidimensional concept closely related to general health status could directly benefit from strength training in older people [24]. A meta-analysis by Hart and Buck [25] reported that after 8 weeks of strength training in older people, HRQoL improved in mental dimensions (including emotional role, mental health, vitality, and social function) and physical dimensions (such as body pain, general health, physical role, and physical function). Consequently, strength training—including body weight–based protocols—has proven effective in promoting physical, psychological, and social well-being in apparently healthy older people [24,26]. Despite this evidence, no systematic review has synthesized experimental studies (randomized or non-randomized controlled trials) evaluating the effects of body weight strength training (BWST) on cognitive function and HRQoL in apparently healthy older people. Addressing this gap could advance understanding in the fields of physical activity and health sciences while providing healthcare professionals with evidence-based guidance on the use of BWST. Therefore, this systematic review aimed to evaluate the available body of published peer-reviewed articles related to the effects of BWST on cognitive function and HRQoL in apparently healthy older people compared with active/inactive control groups.

## 2. Materials and Methods

### 2.1. Protocol and Registration

This review was conducted in accordance with the Preferred Reporting Items for Systematic Reviews and Meta-Analyses (PRISMA) guidelines [27]. The review protocol was prospectively registered in the International Prospective Register of Systematic Reviews (PROSPERO; ID code: CRD42024623038).

### 2.2. Eligibility Criteria

Studies were eligible if they were original, peer-reviewed articles published up to September 2025, without restrictions on language or publication date. Excluded records comprised conference abstracts, book chapters, editorials, letters, protocol registrations, reviews, case reports, and trials not meeting original research criteria. The Population, Intervention, Comparator, Outcome, and Study design (PICOS) framework guided study selection (Table 1).

### 2.3. Information and Database Search Process

The literature search was conducted between November 2024 and September 2025 across six major databases: PubMed/MEDLINE, Web of Science (core collection), Scopus, ScienceDirect, EBSCOhost, and PsycINFO (American Psychological Association). Medical subject headings (MeSH) from the National Library of Medicine of the United States of America use free language terms related to BWST, cognitive function, HRQoL and older people. The search string used was as follows: (“body weight resistance training” OR “body-weight resistance training” OR “body weight” OR “body weight training” OR “calisthenics” OR “resistance training” OR “strength training” OR “high intensity interval training” OR “progressive resistance training” OR “aerobic interval training” OR “high-intensity interval training” OR “moderate-intensity continuous training”) AND (“cognition” OR “cognition functions” OR “executive functions” OR “executive control” OR “cognitive functioning” OR “cognitive control” OR “cognitive functions” OR “cognitive function” OR “memory” OR “cognitive abilities” OR “global cognition” OR “mental flexibility” OR “memory functioning” OR “executive function” OR “short-term memory” OR “long-term memory” OR “cognitive” OR “neurocognition” OR “neurocognitive” OR “neuro-cognition” OR “neuro-cognitive” OR “executive functioning” OR “brain” OR “brain function” OR “brain structure” OR “brain development” OR “cognitive performance” OR “language”) AND (“QoL” OR “HRQoL” OR “quality of life” OR “quality of life perception” OR “health related quality of life” OR “health-related quality of life” OR “mental health” OR “psychological health” OR “body image perception” OR “life satisfaction” OR “lifestyle” OR “healthy lifestyle” OR “psychological well-being” OR “emotional well-being” OR “health status” OR “health status indicators” OR “vitality”) AND (“elderly” OR “older adults” OR “older people” OR “older subject” OR “older participants” OR “aging” OR “ageing” OR “aged”). The lists of eligible and excluded studies were independently reviewed by two experts, both of whom hold doctorates in sports science and have peer-reviewed publications in journals indexed in the Journal Citation Reports^®^ on physical activity or sports science. To avoid bias, the original search strategy was not provided to the reviewers. Following this step, on 11 September 2025, a search was conducted for errata and retractions related to the studies included in the same databases.

### 2.4. Study Selection and Data Collection Process

The studies were managed using Mendeley Reference Manager (version 1.19.8). Two reviewers (Á.L.C. and P.A.-S.) independently performed the literature search, removed duplicates, screened titles and abstracts, and assessed the full texts for eligibility. No discrepancies were found at this stage. The process was repeated for searches within reference lists and suggestions provided by external experts. The full texts of potentially eligible studies were subsequently reviewed, and the reasons for excluding those studies that did not meet the selection criteria were reported.

### 2.5. Methodological Quality Assessment

Study quality was appraised using the TESTEX scale, a 15-point instrument tailored for exercise interventions (5 points for methodological rigor and 10 points for reporting completeness) [29]. Two independent reviewers (Á.L.C. and P.A.-S.) performed the evaluations, while a third author (J.H.-M.) served as a referee for ambiguous situations, subsequently validated by another author (P.V.-B.).

### 2.6. Data Synthesis

Data synthesis consisted of systematically presenting the characteristics and results of the included studies. The variables included publication details (author, year, country), study characteristics (design, baseline health status, sample size, mean age), intervention and comparator information (exercise modality, training volume, intensity), data collection instruments for cognitive function and HRQoL, and main outcomes expressed as means and standard deviations.

### 2.7. Risk of Bias in Individual Studies

Risk of bias was independently evaluated by two reviewers (Á.L.C. and E.V.-C.) using the Cochrane RoB 2 tool as described in the Cochrane Handbook for Systematic Reviews of Interventions [30]. A third reviewer (J.H.-M.) resolved discrepancies. Assessed domains included: randomization process, deviations from intended interventions, missing outcome data, measurement of outcomes, and selective reporting. Each domain for every study was rated as “low risk,” “some concerns,” or “high risk,” and the overall distribution of risk judgments across domains was summarized graphically as percentages [30].

### 2.8. Summary Measures for the Meta-Analysis

The study protocol called for a meta-analysis, the full details of which are available from PROSPERO, registration code: CRD4202462623038. Nevertheless, the limited number of RCTs and the heterogeneity of the instruments used to assess cognitive function and HRQoL made it difficult to perform a meta-analysis.

### 2.9. Certainty of Evidence

The overall certainty of evidence was evaluated using the GRADEpro (Grading of Recommendations, Assessment, Development, and Evaluation) framework, categorizing evidence levels as high, moderate, low, or very low [31]. Since only RCTs were included, the initial rating was high but could be downgraded when concerns arose regarding risk of bias, inconsistency, imprecision, indirectness, or potential publication bias. Two reviewers (Á.L.C. and E.V.C.) independently performed the assessments, and any disagreements were resolved through consensus with a third reviewer (J.H.M.).

## 3. Results

### 3.1. Study Selection

**Figure 1:**
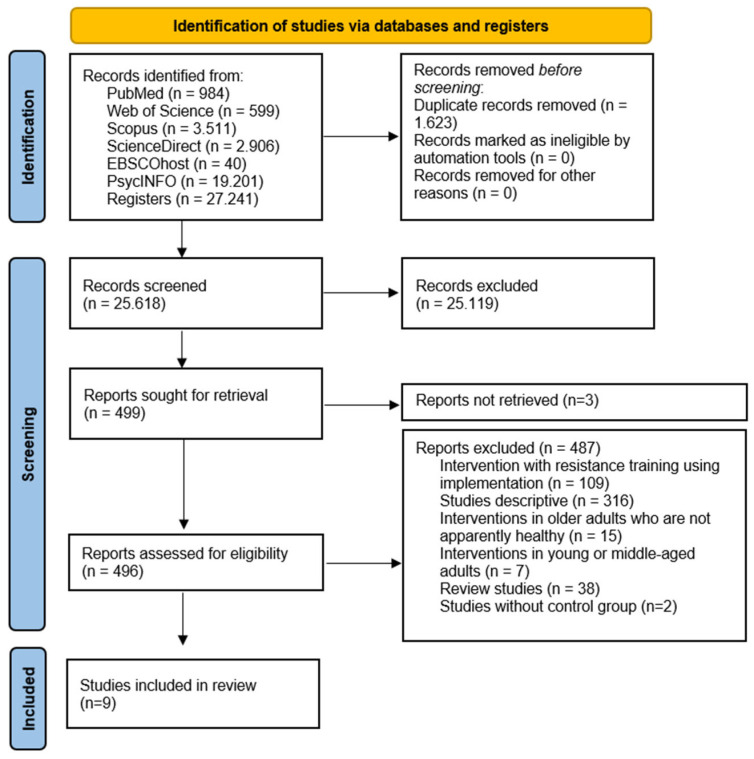
Flowchart of the review process. Legends: Based on the PRISMA guidelines [27].

As illustrated in Figure 1, the initial search yielded 27,241 records. After duplicate removal and title/abstract/keyword screening, 25,618 records remained. Subsequent evaluation excluded 25,119 articles for not meeting the eligibility criteria, leaving 499. Consequently, three studies were excluded because the texts were not accessible (authors of inaccessible studies were contacted and asked for a copy of their manuscript, with an estimated maximum response time of 30 days). In the subsequent analysis phase, 109 strength training intervention studies involving implementation, 316 descriptive studies, 15 intervention studies involving unhealthy older people, 7 studies involving people under 60 years of age, 38 reviews, and 2 studies without a control group were excluded. After this process, nine studies met all the selection criteria [9,20,26,32,33,34,35,36,37].

### 3.2. Methodological Quality

All nine included trials were appraised using the TESTEX instrument (Table 2). All studies achieved a score equal to or above 60% on the scale, namely, 9/15 [9,20,32,33,34], and 11/15 [26,35,36,37], representing at least 60% compliance with the methodological criteria. Therefore, no studies were excluded based on the TESTEX results.

### 3.3. Risk of Bias Within Studies

**Figure 2:**
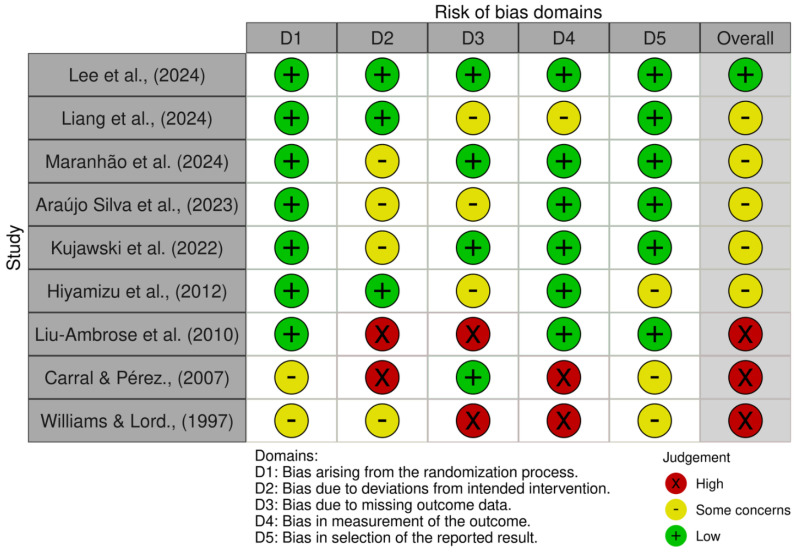
Risk of bias tools: traffic lights chart [9,20,26,32,33,34,35,36,37].

**Figure 3:**
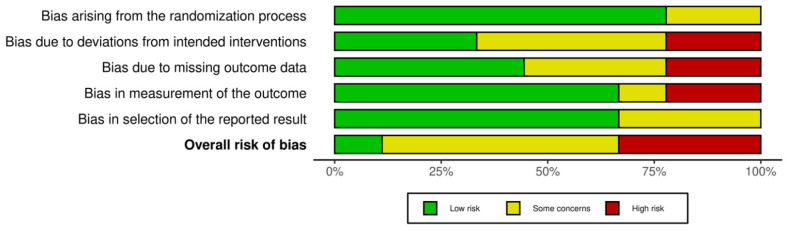
Risk of bias tools: Summary chart by domain.

One study was evaluated as having a minimal risk of bias [34], while four studies exhibited certain issues [9,26,32,36]. Four studies were categorized as possessing a significant risk of bias [20,33,35,37]. This indicates a moderate risk of bias, as the majority of research presented certain problems, while one showed a high risk. Figure 2 and Figure 3 summarize the evaluations of bias risk.

### 3.4. Study Characteristics

The variables analyzed in the nine selected studies are listed in Table 3. One study was conducted in South Korea [34], one in the United Kingdom [35], two in Brazil [26,36], one in Poland [9], one in Japan [32], one in Canada [37], one in Spain [20] and one in Australia [33]. All nine studies were RCTs [9,20,26,32,33,34,35,36,37].

### 3.5. Sample Characteristics

The nine studies presented 27 to 187 participants [9,20,26,32,33,34,35,36,37]. Consequently, the cumulative sample size in all these studies involved 682 (85% female) apparently healthy older people with a mean age of 70 years [9,20,26,32,33,34,35,36,37].

### 3.6. Dosing and Conducted Interventions

Intervention durations ranged from 5 to 42 weeks [9,20,26,32,33,34,35,36,37], with 2 to 7 sessions per week of 10 to 60 min [9,20,26,32,33,34,35,36,37]. The number of exercises ranged from 1 to 4 stimulating large muscle groups of the lower body, such as the quadriceps, abductors, hamstrings, and gastrocnemius [26,32,34,35,36], 1 to 2 exercises stimulating upper body large muscle groups, such as the pectorals and deltoids, and one core exercise [26,32,34,35,36]. Four studies did not report the number of exercises [9,20,33,37]. In addition, 4 studies reported the number of sets and repetitions, ranging from 2 to 3 sets of 8 to 12 repetitions, with a rest 1 min between each set [26,34,36]. One study described the duration of the intervention as a function of time, specifying that each exercise was extended for 1 min with a 1 min rest between exercises [35]. Five studies did not report the number of sets and repetitions [9,20,32,33,37]. On the other hand, one study reported that the intensity of the sessions was assessed via the Borg Perception of Exertion (RPE) scale and the resting heart rate [34], whereas 8 studies did not provide this information [9,20,26,32,33,35,36,37].

Regarding the control groups, 3 studies included active control groups with BWST interventions lasting 12 weeks, ranging from 2 to 3 sessions of 20 to 60 min per week [26,32,36]. Two studies reported 4 exercises for the lower body (sitting to stand, standing hip abduction, pelvic elevation and plantar flexion) and 3 exercises for the upper body (wall push-ups, supine abdominal and unilateral infra-abdominal), with 2 sets of 8 to 10 repetitions and a rest of 1 min between sets [26,36]. In the case of Hiyamizu et al. [32], the control group performed 2 exercises for the lower body (sitting to stand and wall squats using 1 or 2 legs) and 1 exercise for the upper body (wall push-ups), without reporting the number of sets and repetitions. On the other hand, 3 studies reported active control groups using bodybuilding machines, the duration of the interventions ranged from 5 to 52 weeks, with 2 to 3 sessions per week [9,20,37]. In addition, the number of exercises ranged from 2 to 6, stimulating large muscle groups of the lower body, such as quadriceps, hamstrings, gastrocnemius, and adductors, and 3 exercises stimulating large muscle groups of the upper body, such as biceps, triceps, deltoids, latissimus dorsi and pectorals, and 1 core exercise [9,37]. Only one study did not report the number of exercises [20]. Two studies reported the number of sets and repetitions, ranging from 2 to 4 sets of 6 to 15 repetitions [9,37]. Only one study reported a 45 s rest between each set [9]. Meanwhile, one study did not report the number of sets and repetitions [20]. In contrast, 3 studies reported inactive control groups without intervention and continued with their activities of daily living [33,34,35].

### 3.7. Cognitive Function

The selected studies did not qualify for meta-analysis due to the heterogeneity of the assessments used to measure cognitive function. Nevertheless, individual results by Lee et al. [34] reported improvements in orientation to place (*p* < 0.05), language, and visuospatial ability (*p* = 0.02) in favor of the BWST compared with the active control group, whereas attention and calculation only reported within-group improvements in the BWST (*p* = 0.04), as assessed by the Mini-Mental State Examination. Kujawski et al. [9] observed improvements in visual information processing speed in favor of the BWST compared to the active control group (*p* = 0.02) and within-group improvements (*p* = 0.01), as assessed by the Simple Reaction Time, whereas set shifting only reported within-group improvements in the BWST (*p* = 0.04), as assessed by the Trail Making test. Hiyamizu et al. [32] reported improvements in attention in favor of the BWST compared with the active control group (*p* = 0.04), as assessed by the Stroop test with a dual task. Carral and Pérez [20] reported within-group improvements in the general cognitive function score in both the BWST (*p* = 0.03) and active control group (*p* = 0.02), as assessed by the Mini-Mental State Examination. Williams and Lord [33] reported improvements in short-term memory in favor of the BWST (*p* = 0.02) compared with the inactive control group, as assessed by the Digit Span test of the Wechsler Adult Intelligence Scale-Revised. On the other hand, Maranhão et al. [36] reported improvements in inhibitory control (*p* = 0.03) in favor of the active control group compared to the BWST as assessed by the Stroop test. Meanwhile, Liu-Ambrose et al. [37] reported a decrease in selective attention and conflict resolution in the BWST compared to active control groups (*p* < 0.05) as assessed by the Stroop test.

### 3.8. Health-Related Quality of Life (HRQoL)

The selected studies did not qualify for meta-analysis because of the heterogeneity of the assessments used to measure HRQoL. However, Daniele de Araújo Silva et al. [26] reported improvements in the dimensions of past, present, and future activities, social participation, intimacy, and general score of HRQoL in favor of the active control group compared to the BWST (*p* < 0.05), as assessed by the World Health Organization Quality of Life questionnaire for older people. On the other hand, Carral and Pérez [20] reported within-group improvements in components related to HRQoL, specifically health-related anxiety, in both the BWST (*p* = 0.04) and in the active control group (*p* = 0.04), as assessed by the Health Orientation Scale. In contrast, Liang et al. [35] did not observe significant differences between the BWST and the inactive control group in the dimensions of physical health (*p* = 0.28), mental health (*p* = 0.38), and general (*p* = 0.68) HRQoL scores, as assessed by the five-dimensional, five-level EuroQol questionnaire and the Short Form Health Survey-36.

### 3.9. Certainty of Evidence

The GRADE evaluation of the available trials indicated that, although the intervention demonstrated benefits in cognitive function and HRQoL, it was not possible to precisely estimate the relative effect. The certainty of evidence was rated as moderate, primarily limited by concerns about risk of bias, while consistency, applicability, and precision did not raise major concerns. Overall, these findings are considered clinically relevant and support the value of the intervention, yet they underscore the need for future studies with more rigorous designs to confirm and quantify its true effect (Table 4).

### 3.10. Adherence and Adverse Events

The studies reported adherence rates of 100% [20], 90% [35], 72% [33], 62% [37] and 60% [26,36]. Three studies did not report with BWST [9,32,34].

Regarding adverse events, only one study reported musculoskeletal complaints in 9.5% of participants and one fall without injury during the intervention with BWST [37]. Meanwhile, 6 studies reported adverse events unrelated to the intervention with BWST, including stroke, death, falls [33] and other health problems [9,26,32,33,35,36]. Two studies reported no adverse events during the intervention with BWST [20,34]. On the other hand, the main reasons for dropping out were personal problems, health issues, failure to meet the minimum attendance requirements for training sessions, and withdrawal of informed consent [9,26,32,33,34,35,36,37]. Only one study reported that none of the participants dropped out of the intervention with BWST [20].

## 4. Discussion

This systematic review aimed to evaluate the available body of published peer-reviewed articles related to the effects of BWST on cognitive function and HRQoL in apparently healthy older people compared with active/inactive control groups. After 27,241 records were reviewed, 9 RCTs involving 682 (85% women) older people were included. All selected studies scored 60% or higher on the TESTEX methodological scale. The heterogeneity observed in the assessments to measure cognitive function and HRQoL limited the possibility of performing a meta-analysis. Nevertheless, the results of the individual studies indicate that, compared with the active/inactive control groups, the BWST resulted in significant improvements in orientation to place, language, visuospatial ability, processing speed, attention, and short-term memory. Within-group improvements were observed in general cognitive function and health-related anxiety scores in both the BWST and active control groups. The calculation and set shifting improved only within the BWST group. In contrast, inhibitory control, the dimensions of past, present, and future activities, social participation, intimacy, and general HRQoL scores improved in the active control group.

### 4.1. Cognitive Function

The results of the studies analyzed reported moderate certainty of evidence with significant improvements in different areas of cognitive function in the BWST [9,20,32,33,34]. These findings are consistent with the study by Sanchez-Lastra et al. [19] reported that institutionalized older people improved their general cognitive function score (*p* = 0.008) and attention (*p* = 0.031) after 12 weeks of upper and lower body strength training. Similarly, the study by Coetsee and Terblanche [10] in apparently healthy older people after 16 weeks physical training in three different modalities (strength, moderate continuous aerobic, and high-intensity aerobic interval), reported that the strength training group achieved greater within-group improvements in processing speed compared with the moderate continuous aerobic training group (15.05%, ES = 1.00 vs. 6.33%, ES = 0.69; *p* < 0.05), and greater within-group improvements in executive function compared to the high-intensity aerobic interval training group (48.43%, ES = 1.07 vs. 20.85%, ES = 0.45; *p* < 0.05), as assessed by the Stroop test. However, a meta-analysis by Xiong et al. [13] in apparently healthy older people reported that aerobic training results in better executive function, specifically working memory (Hedge’s g = 0. 098, 95% CI = 0.017–0.178; *p* = 0.017, I^2^ = 0%), inhibitory control (Hedge’s g = 0.136; 95% CI = 0.053–0.218; *p* = 0.001, I^2^ = 0%), and cognitive flexibility (Hedge’s g = 0.566; 95% CI = 0.135–0.997; *p* = 0.010, I^2^ = 0%) compared with strength and mind–body training, results that were primarily assessed by the Digit Span test, N-back task, Task switching, Trail making test, Wisconsin card sorting test, and Stroop test. Both types of training are highly effective in improving cognitive function because of their ability to increase blood flow and the oxygen supply to brain tissue, preventing the neurodegenerative process associated with aging [14]. Strength training stands out for requiring a lower weekly exercise dose (529 METs-min; 3 sessions of 40 min of moderate intensity without considering warm-up and cool-down) to obtain clinically significant benefits in cognitive function compared with aerobic activities (758 METs-min; 4 sessions of 45 min of moderate intensity without considering warm-up and cool-down) [15], thus aligning with the minimum international physical activity recommendations (600 METs-min) for healthy brain aging [6,11].

Furthermore, strength training combined with cognitive training (simultaneous dual-task training) in apparently healthy older people also yielded promising results. Hiyamizu et al. [32] reported significant improvement in attentional cognitive function after 12 weeks of BWST combined with cognitive tasks of calculation, visual search and verbal fluency compared with the control group performing only BWST (*p* = 0.04). Similarly, Castano et al. [12] reported that after 16 weeks of strength training combined with a verbal fluency cognitive task (2 sessions of 60 min per week), in which older people were required to mention aloud as many words as possible from a specific category (e.g., countries, colors, transportation) in each set of exercises, improved verbal fluency and short-term memory (*p* < 0.05). In contrast to the group that only performed conventional strength training without obtaining better results in these variables, according to the Scenery Picture Memory, semantic, and phonological tests [12]. These findings suggest that the BWST [20,33] combined with simultaneous cognitive training [32], could induce changes in brain neuroplasticity mediated by brain-derived neurotrophic factor, leading to significant improvements in physical performance and cognitive function in apparently healthy older people [12,38,39].

### 4.2. Health-Related Quality of Life (HRQoL)

The results of the studies analyzed reported moderate certainty of evidence with significant improvements in components associated with HRQoL [20,26]. Similarly, a study by Juesas et al. [40] in apparently healthy older people reported that, compared to inactive control group, the strength training group had improvements in dimensions of social function (*p* < 0.001), mental health (*p* = 0.009), vitality (*p* < 0.001), and general health scores (*p* < 0.001) of HRQoL, as assessed the SF-36 questionnaire after 16 weeks of strength training intervention. Likewise, a meta-analysis by Khodadad Kashi et al. [24] in apparently healthy older people reported that strength training improved in dimensions of physical function (SMD = 0.31, 95% CI = 0.04–0.57; *p* = 0.02), mental health (SMD = 0.44; 95% CI = 0.17–0.71; *p* = 0.001; I^2^ = 66%; *p* < 0.0001), body pain (SMD = −0.52; 95% CI = −0.87–−0.16; *p* = 0.004), general health (SMD = 0.43; 95% CI = 0.16–0.70; *p* = 0.002), and social function (SMD = 0.25; 95% CI = 0.07–0.42; *p* = 0.006) of HRQoL, results obtained mainly from the SF-36, SF-12, SF-8, WHOQOL-BREF, and WHOQOL-OLD assessments. In turn, reports improved upper (MD, 15.26 kg; 95% CI, 5.51–25.00; *p* = 0.002) and lower (MD, 48.46 kg; 95% CI, 6.53–90.39; *p* = 0.02) body muscle strength [24]. These results may be due to an association between HRQoL and physical fitness. Valdés-Badilla et al. [23] noted that apparently healthy older people with low performance in lower (OR = 1.11; *p* = 0.001) and upper (OR = 1.07; *p* = 0.003) body muscle strength tests had an increased probability of having a low HRQoL in the physical function dimensions. Similarly, Haraldstad et al. [41] reported that 12 weeks of strength training increases lower body muscle strength and upper body lean muscle mass, which are positively correlated with the physical function (*p* = 0.042) and social function (*p* = 0.021) dimensions of HRQoL, as assessed by the SF-12 questionnaire. However, Liang et al. [35] reported nonsignificant effects on HRQoL after BWST intervention. This could be because the heterogeneity of the instruments used may not be sensitive enough to detect relevant changes in HRQoL after an intervention with BWST in apparently healthy older people. Nevertheless, unconventional physical activity strategies with minimal implementation demonstrate a mean adherence rate >80% and a small (*d* = 0.49) and moderate (*d* = 0.72) positive effect size on HRQoL in healthy middle-aged and older people [42]. Therefore, the BWST could be a cost-efficient alternative, allowing us to counteract risk factors associated with loss of muscle strength, anxiety and depressive symptoms and improving HRQoL in apparently healthy older people [18,20,26].

On the other hand, certain active control groups achieved significant improvements in components of cognitive function and HRQoL compared to the BWST [26,36,37]. These results should be taken with caution because they may be influenced by various factors such as the participants’ previous experience with strength training, which could yield minimal, non-significant benefits in cognitive function and HRQoL [15,25]. While BWST in these studies [26,36,37] was not designed to achieve large gains in muscle strength, but rather to maintain the functional independence of older people, which could impact potentially significant improvements in cognitive function and HRQoL [43]. Furthermore, baseline values for cognitive function and HRQoL close to maximum limit the observation of significant room for improvement [26,36,37,44].

### 4.3. Limitations and Strengths

The main limitations of this systematic review include the following: (i) the small number of RCTs and the diversity of instruments used to assess cognitive function and HRQoL, which makes it difficult to perform a meta-analysis of the data; (ii) most of the instruments used to assess cognitive function were originally designed for the clinical diagnosis of cognitive disorders, rather than to assess normative performance [11]; (iii) reliance on self-reported measures may have influenced the observed effects, as their validation does not eliminate susceptibility to expectation or placebo biases, particularly in BWST interventions, potentially leading to an overestimation of perceived improvements; (iv) most RCTs included small samples composed predominantly of women and recruitment processes that may have attracted highly motivated participants expecting positive outcomes, introducing potential selection bias, reducing the certainty of evidence, and limiting the generalizability of the findings; (v) the HRQoL evidence is less consistent, as only three RCTs provided data and, in some cases, results favored the control group, limiting the strength of conclusions in this domain; (vi) individual results should be interpreted with caution, given that intervention intensity was not consistently reported, while exercise volume, intensity, and type varied considerably, making comparisons inconclusive; (vii) the optimal BWST dosage to maximize cognitive and HRQoL benefits remains unclear; (viii) adherence rates were relatively low in some trials (ranging from 62% to 72%), which may have affected internal validity; and (ix) heterogeneity in control group designs may have contributed to the insufficient certainty of evidence to establish definitive recommendations on BWST, as inactive controls in RCTs are less effective in accounting for placebo or expectation effects, particularly when combined with self-reported outcomes for cognitive function and HRQoL. On the other hand, the strengths of the studies included the following: (i) methodological quality above 60% in the reviewed studies; (ii) methodological procedures of the PRISMA, PROSPERO, TESTEX, RoB 2 and GRADE scales; (iii) use of a core collection of six generic databases (PubMed, Web of Science, Scopus, ScienceDirect, EBSCOhost, PsycINFO); and (iv) analysis of how BWST affects cognitive function and HRQoL in apparently healthy older people.

### 4.4. Practical Applications

The results of this systematic review suggest that BWST may have positive effects on cognitive function, especially in higher-level cognitive domains such as executive functions, and on HRQoL. According to the findings, a weekly frequency of 2–3 sessions of 20–60 min aligns with previous meta-analysis on the effects of strength training on cognitive function [15], HRQoL [24], and physical performance [18] in apparently healthy older people. Moreover, BWST represents a promising intervention due to its accessibility and low economic cost, making it a viable and easily applicable strategy for physical activity and health professionals in community or home-based settings for apparently healthy older people [43,45].

### 4.5. Future Research Directions

It is recommended that upcoming RCTs incorporate more rigorous methodological designs and objective cognitive function assessments complementary to self-reported measures [11,43], as well as active control groups that minimize potential expectation or placebo biases to yield more accurate results [44]. Furthermore, greater adherence to BWST programs should be encouraged, which could be achieved through behavioral and motivational strategies such as individualized goal setting, the use of remote monitoring technologies (mobile applications or wearable devices) [36], and the implementation of group-based programs that foster social interaction. Replication of findings in high-quality RCTs will contribute to consolidating the evidence on the effects of BWST on cognitive function and HRQoL in apparently healthy older people [44]. Finally, it is necessary to establish optimal dosage parameters for BWST interventions to ensure consistent benefits for cognitive function and HRQoL. In this regard, the Consensus on Exercise Reporting Template (CERT) provides a standardized framework comprising 16 essential elements that facilitate detailed descriptions of strength training principles, including frequency, intensity, time, and type of exercise implemented in RCTs [43,46].

## 5. Conclusions

The individual results of the RCTs suggest that BWST may have potential effects on cognitive function and HRQoL in apparently healthy older people. Nevertheless, the limited number of available RCTs and the observed methodological heterogeneity reduce the certainty of evidence, making it insufficient to support definitive recommendations. BWST appears to be a promising, feasible, and safe intervention for this population. Therefore, further high-quality studies are needed to establish robust conclusions and confirm the preliminary findings.

## Figures and Tables

**Table 1:** Selection criteria used in the systematic review.

Category	Inclusion Criteria	Exclusion Criteria
Population	Apparently healthy older people with a mean age of 60 years or more according to the World Health Organization [28], and without distinction of sex.	People under 60 years of age.
Intervention	Intervention with BWST (i.e., calisthenics, HIIT, multicomponent training) lasting four weeks or more.	Interventions that do not include BWST.
Comparator	Interventions with a control group with or without supervised physical activity.	Absence of control group.
Outcomes	At least one cognitive function assessment (i.e., Mini-mental, Montreal Cognitive Assessment, Stroop test) and/or HRQoL assessment (i.e., SF-36, SF-12, WHOQOL-OLD).	Lack of baseline and/or follow-up data.
Study design	Experimental design studies (randomized controlled trial and nonrandomized controlled trial) with pre- and post-assessment.	Cross-sectional, retrospective, and prospective studies.

BWST: Body weight strength training; HIIT: High-intensity interval training; HRQoL: Health-related quality of life; SF-12: Short Form Health Survey-12; SF-36: Short Form Health Survey-36; WHOQOL-OLD: World Health Organization Quality of Life questionnaire for older people.

**Table 2:** Methodological quality assessment of the studies according to the TESTEX scale.

Study	Eligibility Criteria Specified	Randomly Allocated Participants	Allocation Concealed	Groups Similar at Baseline	Assessors Blinded	Outcome Measures Assessed >85% of Participants *	Intention to Treat Analysis	Reporting of Between-Group Statistical Comparisons **	Point Measures and Measures of Variability Reported	Activity Monitoring in Control Group	Relative Exercise Intensity Reviewed	Exercise Volume and Energy Expended	Overall TESTEX #
Liang et al. [35]	1	1	1	1	0	2/3	1	2/2	1	0	1	0	11/15
Lee et al. [34]	1	1	1	1	0	1/3	0	2/2	1	0	0	1	9/15
Maranhão et al. [36]	1	1	1	1	1	1/3	1	2/2	1	0	1	0	11/15
Daniele de Araújo Silva et al. [26]	1	1	1	1	1	1/3	1	2/2	1	0	1	0	11/15
Kujawski et al. [9]	1	1	1	1	0	1/3	0	2/2	1	0	1	0	9/15
Hiyamizu et al. [32]	1	1	0	1	1	2/3	0	2/2	1	0	0	0	9/15
Liu-Ambrose et al. [37]	1	1	1	1	1	2/3	1	2/2	1	0	0	0	11/15
Carral & Pérez [20]	1	1	1	1	0	2/3	0	2/2	1	0	0	0	9/15
Williams & Lord [33]	1	1	1	1	0	2/3	0	2/2	1	0	0	0	9/15

* Three points are possible: one point if adherence >85%, one point if adverse events were reported, and one point if exercise attendance was reported. ** Two points possible: one point if the primary outcome is reported and one point if all other outcomes are reported. # Total out of 15 points. TESTEX: Tool for assessing study quality and reporting in exercise [29].

**Table 3:** Studies report the effects of body weight strength training on cognitive function and health-related quality of life in apparently healthy older people.

Study	Country	Study Design	Sample’s Initial Health	Groups	Mean (Age, Years)	Type of Interventions and Control Groups	Training Volume			Training Intensity	Cognitive Function	HRQoL	Main Outcomes
							Weeks	Frequency (Sessions/Weeks)	Session Duration (min)				
Lee et al. [34]	SK	RCT	Apparently healthy	BWST: 13 CG: 14 (63% of female and 37% of male)	BWST: 70.6 ± 3.14 CG: 71.5 ± 5.93	BWST: upper and lower body exercises without external load. CG: no intervention continued with their daily activities.	8	3	40–50	RPE (1–10) and HR	MMSE (score): Orientation to time, Orientation to place, Memory registration, Attention and calculation, Memory recall, Language and visuospatial ability	NR	Cognitive function BWST vs. CG ↑ Orientation to place ↑ Language and visuospatial ability ↔ Orientation to time ↔ Memory registration ↔ Memory recall ↔ General score Only BWST ↑ Attention and calculation
Liang et al. [35]	UK	RCT	Apparently healthy	BWST: 44 CG: 46 (71% of female and 29% of male)	BWST: 74.0 ± 5.5 CG: 74.2 ± 5.6	BWST: upper and lower body exercises without external load. CG: no intervention continued with their daily activities.	12	7	10	NR	NR	EQ-5D-5L (score) SF-36 (score): Physical health and Mental health	HRQoL BWST vs. CG ↔ General score (EQ-5D-5 L) ↔ Physical health (SF-36) ↔ Mental health (SF-36)
Maranhão et al. [36]	BR	RCT	Apparently healthy	BWST: 18 CG: 20 (82% of female and 18% of male)	BWST: 68 ± 5.88 CG: 69 ± 7.05	BWST: upper and lower body exercises without external load and virtual supervision. CG: upper and lower body exercises without external load and minimal supervision.	12	3	20-30	NR	TMT-A: Processing speed (s) TMT-B: Processing speed (s) ST: Inhibitory control (interference score) SVF: Verbal fluency (score)	NR	Cognitive function BWST vs. CG ↔ TMT-A ↔ TMT-B ↔ SVF Only CG ↑ ST
Daniele de Araújo Silva et al. [26]	BR	RCT	Apparently healthy	BWST: 18 CG: 20 (82% of female and 18% of male)	BWST: 68 ± 5.88 CG: 69 ± 7.05	BWST: upper and lower body exercises without external load and virtual supervision. CG: upper and lower body exercises without external load and minimal supervision.	12	3	20-30	NR	NR	WHOQOL-OLD (score): Sensory skills, Autonomy, Past, present, and future activities, Social participation, Intimacy, Death and dying	HRQoL BWST vs. CG ↑ General score HRQoL ↑ Past, present, and future activities ↑ Social participation ↑ Intimacy ↔ Sensory skills Autonomy ↔ Death and dying
Kujawski et al. [9]	PL	RCT	Apparently healthy	BWST: 28 CG: 27 (93% of female and 7% of male)	BWST: 67.7 ± 6 CG: 64.6 ± 4	BWST: upper body exercises without external load. CG: upper and lower body exercises with bodybuilding machines.	12	2	45	NR	MoCA (score) TMT-B: Set-shifting (s) DS: Attention (score) Battery Test Sprawności Operacyjnej: SRT, CRT, VAT and DMS	NR	Cognitive function BWST vs. CG ↔ MoCA ↔ TMT-B ↔ DS ↑ SRT ↔ CRT ↔ VAT Only BWST ↑ TMT-B ↑ SRT Only CG ↑ MoCA ↑ SRT ↑ CRT ↑ VAT
Hiyamizu et al. [32]	JP	RCT	Apparently healthy	BWST: 17 CG: 19 (72% of female and 28% of male)	BWST: 72.9 ± 5.1 CG: 71.2 ± 4.4	BWST: upper and lower body exercises without external load with dual tasks. CG: upper and lower body exercises without external load.	12	2	60	NR	ST: Attention (score) TMT-A: Processing speed (s) TMT-B: Set shifting (s) TMT B-A: Attention (s)	NR	Cognitive function BWST vs. CG ↑ ST ↔ TMT-A ↔ TMT-B ↔ TMT B-A
Liu-Ambrose et al. [37]	CA	RCT	Apparently healthy	BWST: 49 CG1: 54 CG2: 52 (100% of female)	BWST: 70 ± 3.3 CG1:69.5 ± 2.7 CG2: 69.4 ± 3.0	BWST: upper body exercises without external load. CG1: upper and lower body exercises with bodybuilding machines. CG2: upper and lower body exercises with bodybuilding machines	52	2	60	NR	ST: Selective attention and conflict resolution (s) TMT B-A: Set shifting (s) DSF-DSB: Working memory (score)	NR	Cognitive function BWST vs. CG ↓ ST ↔ TMT B-A ↔ DSF-DSB
Carral & Pérez [20]	ES	RCT	Apparently healthy	BWST: 27 CG: 29 (100% of female)	BWST: 68.5 ± 3.4 CG: 68.3 ± 3.49	BWST: upper and lower body exercises without external load. CG: upper and lower body exercises with bodybuilding machines	5	3	45	NR	MMSE (score)	HOS (score): Interest in health, Health image concern, Internal health locus of control, Personal health consciousness, Health-related anxiety	Cognitive function BWST and CG ↑ General score (MMSE) HRQoL BWST1 and CG ↑ Health-related anxiety Only CG ↑ Internal health locus of control Interest in health ↔ Health image concern ↔ Personal health consciousness
Williams & Lord [33]	AU	RCT	Apparently healthy	BWST: 94 CG: 93 (100% of female)	BWST: 71.8 ± 5.6 CG: 71.6 ± 5.2	BWST: upper and lower body exercises without external load CG: no intervention continued with their daily activities.	42	2	60	NR	DS (WAIS-R): Short-term memory (score) PA (WAIS-R): Nonverbal reasoning ability (score) CM: Nonverbal reasoning ability and Problem solving (score)	NR	Cognitive function BWST vs. CG ↑ DS (WAIS-R) ↔ PA (WAIS-R) ↔ CM

↑: Significant increase; ↓: Significant decrease; ↔: No significant differences; AU: Australia; BR: Brazil; BWST: Body weight strength training; CA: Canada; CG: Control group; CG1: Control group 1; CG2: Control group 2; CM: Cattell’s Matrices Test; CRT: Choice Reaction Time; DS: Digit Span Test; DSB: Digit Span Backward; DSF: Digit Span Forward; DS (WAIS-R): Digit Span (Wechsler Adult Intelligence Scale-Revised); DMS: Delayed Matching to Sample; EQ-5D-5L: EuroQoL five-dimension, five-level questionnaire; ES: Spain; HOS: Health Orientation Scale; HR: Heart rate; HRQoL: Health-related quality of life; JP: Japan; MMSE: Mini-Mental State Examination; MoCA: Montreal Cognitive Assessment; NR: Not reported; PA (WAIS-R): Picture Arrangement (Wechsler Adult Intelligence Scale-Revised); PL: Poland; RCT: Randomized controlled trial; RPE: Rate of perceived exertion—Borg 10 scale; SF-36: Short Form Health Survey-36; SVF: Semantic Verbal Fluency of Animals Test; SK: South Korea; SRT: Simple Reaction Time; ST: Stroop Test; ST: Stroop Test; TMT: Trail Making Test; TMT-A: Trail Making Test A; TMT-B: Trail Making Test B; TMT B-A: Trail Making Test B-A; VAT: Visual Attention Test; WHOQOL-OLD: World Health Organization Quality of Life questionnaire for older people.

**Table 4:** Methodological quality assessment via the GRADEpro tool.

Certainty Assessment							Number of Patients		Effect		Certainty	Importance
Number of Studies	Study Design	Risk of Bias	Inconsistency	Indirect Evidence	Vagueness	Other Considerations	[Intervention]	[Comparison]	Relative (95% CI)	Absolute (95% CI)		
Cognitive function												
6	RCT	Serious to	It is not serious	It is not serious	It is not serious	None	219/498 (44.0%)	279/498 (56.0%)	Not estimable		⨁⨁⨁ ◯ Moderate to	IMPORTANT
HRQoL												
3	RCT	Serious to	It is not serious	It is not serious	It is not serious	None	89/184 (48.4%)	95/184 (51.6%)	Not estimable		⨁⨁⨁ ◯ Moderate to	IMPORTANT

RCT: Randomized controlled trial. HRQoL: Health-related quality of life. CI: Confidence interval. ⨁⨁⨁ ◯: Certainty of evidence moderate.

## Data Availability

The datasets generated during and/or analyzed during the current review are available from the corresponding author upon reasonable request.
